# Radiotherapy for cancer using X-ray fluorescence emitted from iodine

**DOI:** 10.1038/srep43667

**Published:** 2017-03-02

**Authors:** Masato Tamura, Hiromu Ito, Hirofumi Matsui

**Affiliations:** 1 Faculty of Medicine, University of Tsukuba, 1-1-1 Ten-noudai, Tsukuba, Ibaraki, 305-8573, Japan

## Abstract

Radiation treatment is popular and the apparatus is already available in many hospitals. Conventional radiation treatment by itself is not sufficient to achieve complete cure. Therefore, radiosensitizers have been developed to enhance the therapeutic effects of the treatment. The concept of radiosensitization with high-Z-elements was first considered many decades ago. However, radiosensitizers are not commonly used in the clinical setting. Here, we propose a radiotherapy method that utilizes fluorescent X-ray emissions from iodine. This approach should achieve a greater therapeutic effect than that of conventional radiotherapy treatments. In our radiotherapy, iomeprol was used as the iodine-donor. The X-ray apparatus with copper and aluminum filters could be used for the X-ray irradiation, the apparatus is not needed for exclusive use. The X-ray apparatus is only required to prepare the copper and aluminum filters. As proof-of-concept, we show that tumor growth was attenuated using this treatment with iomeprol.

Surgery is not always considered in a cancer treatment plan; the patient may have to be re-admitted and surgery would decrease the patient’s quality-of-life. This is especially true for elderly patients^1^. These patients may be treated by radiation therapy. Radiotherapy options such as boron neutron capture therapy and heavy ion radiotherapy represent the most advanced medical radiation treatment options, although these treatments can only be performed in larger facilities^2^,^3^,^4^. Although radiation treatment is popular and the apparatus is already available for use in many hospitals, the conventional radiation treatments cannot achieve a complete response. If therapeutic effects of radiotherapy is improved, we can become to treat large numbers of patient effectively. Use of radiosensitizers are one of strategies to enhance the radiotherapy.

The concept of radiosensitization using elements with a high atomic number Z (high-Z-elements) was introduced many decades ago^5^. The X-ray absorption edge is intrinsic to each atom. The energies of high-Z-elements and oxygen (water) are quite different from each other. Water absorbs X-rays of less than 20 keV well. The human body contains a lot of water; therefore, radiation treatment induces injury. The X-ray absorption edge from a high-Z-element and that from water are different. Therefore, use of the X-ray absorption edge from a high-Z-element for radiation treatment is suitable. X-rays with higher energy than the K-edge of iodine (33.2 keV) are required for fluorescent X-ray emission from iodine^6^. The irradiation can be generated using two-layer metal filters containing copper and aluminum. X-ray less than 31 KeV is attenuated less than 0.0001 fold by through copper filter (1 mm thick). When X-ray through the filter, copper emits fluorescent X-ray including Kα and Kβ line (approximately 8 keV). Next, aluminum filter (2 mm thick) absorbs the fluorescent X-ray from copper filter, resulting in the incident X-ray including negligible X-ray less than 31 keV^7^. Additionally, a high-Z-element-containing drug such as iomeprol can be injected into patients as a contrast agent in computed tomography (CT)^8^.

Fluorescent X-rays including the Kα line (28.5 keV) and L-band (3.78–5.18 keV) energy bands are released from iodine. Radiation injury is sustained only in the local area. Some reports have indicated that iodine functions as a radiosensitizer in both cellular and animal models^9^. Additionally, in clinical trials, administration of iopamidol to patients with metastatic brain tumors enhanced the therapeutic effects of the radiation treatment, and abolished the side effects. In these trials, the irradiation was performed using a CT scanner^10^. Thus, the combination of an iodine-containing drug and irradiation with filters consisting of copper and aluminum plates should result in effective radiation therapy.

In a previous study, we used two cell lines: a green fluorescent protein (GFP)-expressing rat normal gastric mucosal line (RGM-GFP) and a kusabira orange-expressing RGM cancer cell line (RGK-KO)^11^. RGK cells were chemically transformed from RGM cells *in vitro*^12^; therefore, these cells have the same genetic background. These cell lines were used to determine the drug response, toxicities, and side effects^11^.

To show proof-of-concept, we proposed a cancer treatment with radiotherapy using X-ray fluorescence from iodine, namely, a fluorescent X-ray treatment.

## Methods

### Materials

Penicillin/streptomycin, Dulbecco’s Modified Eagles Medium (DMEM), and DMEM/Ham’s F12 were purchased from Thermo Fisher Scientific Inc. (Kanagawa, Japan). Iomeprol (300 mgI/mL in saline) was purchased from Eisai Co., Ltd., Tokyo, Japan, and G418 was purchased from Merck KGaA, Darmstadt, Germany. Iomeprol contains 300 mg/mL of iodine; 612.4 mg/mL iomeprol is equivalent to 300 mgI/mL of iomeprol.

### Cell culture

Rat GFP-expressing gastric mucosal cells (RGM-GFP) and kusabira orange-expressing cancerous RGM cells (RGK-KO) were used in this study^11^. RGM-GFP cells were cultured in DMEM/Ham’s F12 medium, and RGK-KO cells were cultured in DMEM with 10% v/v fetal bovine serum and 1% v/v penicillin/streptomycin. G418 was added to all media, with the exception of that used in cytotoxicity assays. A co-culture with RGM-GFP and RGK-KO cells was used for all cell culture experiments.

### Animals

Seven week-old Crl:CD1 (ICR) and CAnN.Cg-Foxn1^nu^/CrlCrlj (BALB/c-nu) mice were purchased from Charles River Laboratories Japan, Inc. (Kanagawa, Japan). This animal study was approved by the animal care and use committee of the University of Tsukuba (No. 15-102), and was performed according to the permitted study plan.

### Radiation treatment *in vitro*

Fluorescence-based co-cultures with normal and cancer cells were used^11^. RGM-GFP and RGK-KO cells were cultured in 96-well plates at a density of 31,250 cells/cm^2^, with equal numbers for each cell line. After incubation for 24 h, radiation treatment was performed (MBR-1520R, Hitachi, Power Solutions Co. Ltd., Ibaraki, Japan) until the irradiation doses reached 0, 10, and 20 Gy through copper (1 mm thick) and aluminum (2 mm thick) filters. The applied voltage was 150 kV. The distance between the radiation source and the dish was approximately 40 cm. Irradiated sample was put within the X-ray irradiated area under the condition. The sample rotated on a turntable while X-ray irradiation.

The surface areas of the RGM-GFP and RGK-KO cells were measured as pixels using ImageJ 1.45 s software ver.1.6.0 (National Institute of Health, USA) for 10 days.

### Evaluation of radiation injury *in vivo*

ICR mice were irradiated with 7.5 Gy through filters consisting of successive copper and aluminum plates. The heads of the mice were protected by a lead plate. Irradiated mice were monitored for 2 weeks and the survival rate was calculated.

### Evaluation of fluorescent X-ray treatment *in vivo*

RGK-KO cells (1 × 10^6^ cells) were transplanted from a dish to the right legs of BALB/c-nu mice. The tumors were allowed to grow to over 100 mm^2^ for 1–2 weeks, and then iomeprol was injected directly into the tumors of tumor-bearing mice. Irradiation was performed after the injection for 1 h. Tumor size and body weight were measured for 14 days.

## Results

### Radiation treatment to cells with a precise X-ray band

**Figure 1:**
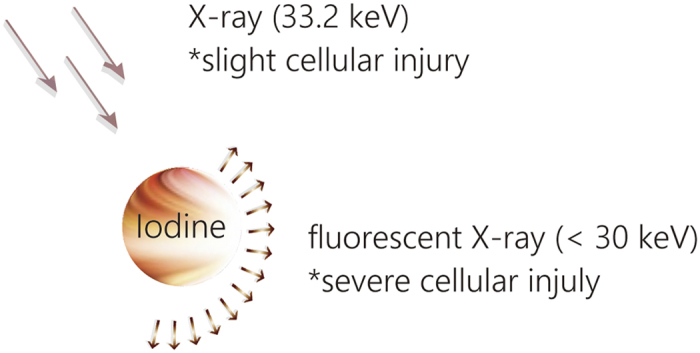
Schematic of the proposed therapy. Irradiation of iomeprol produces fluorescent X-rays.

An X-ray at 33.2 keV becomes a fluorescent X-ray at <30 keV through an excitation of iodine (Fig. 1). Theoretically, X-ray irradiation induces radiotoxicity through the skin to the tumor; however, fluorescent X-ray emissions from iodine induce severe radiotoxicity in the tumor tissue.

**Figure 2:**
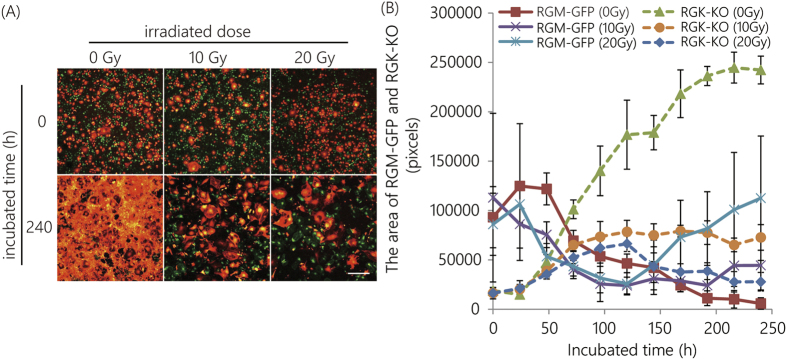
Tumor-specific toxicity after irradiation. A fluorescent co-culture system with both normal (RGM-GFP) and cancerous cells (RGK-KO) was used. (**A**) Fluorescence images. Scale bar indicates 500 μm. (**B**) The area covered by RGM-GFP and RGK-KO cells. Error bars represent standard deviation.

To evaluate this treatment, a co-culture system with RGM-GFP and RGK-KO cells irradiated at 0, 10, and 20 Gy was used. Increasing the radiation dose resulted in tumor cell-specific cell death (Fig. 2). The surface areas covered by RGM-GFP and RGK-KO cells changed accordingly. This result indicated that irradiation (>10 Gy) with copper and aluminum filters could induce cell death.

### Iomeprol treatment

**Figure 3:**
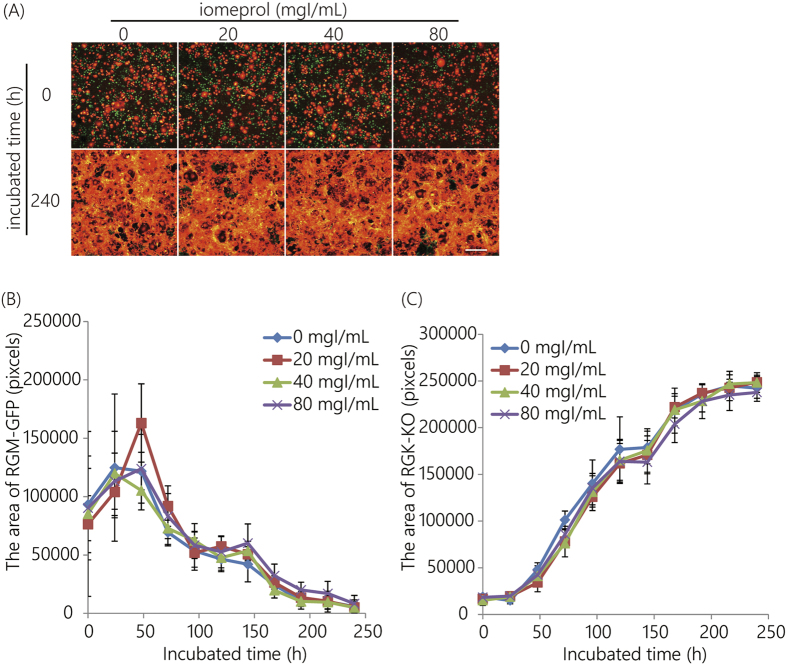
Cell viability assay following iomeprol exposure. A fluorescent co-culture system with both RGM-GFP and RGK-KO cell lines was used. (**A**) Fluorescence images. Scale bar indicates 500 μm. (**B**) The area covered by RGM-GFP cells. (**C**) The area covered by RGK-KO cells. Error bars represent the standard deviation.

The toxicities of iomeprol were evaluated (Fig. 3). Iomeprol doses between 0 and 80 mgI/mL did not cause cell death. These results indicated that RGM-GFP cell numbers decreased and RGK-KO cell numbers increased over time, regardless of whether or not an iomeprol dose of <80 mgI/mL was used (Fig. 3).

### X-ray fluorescence treatment *in vitro*

**Figure 4:**
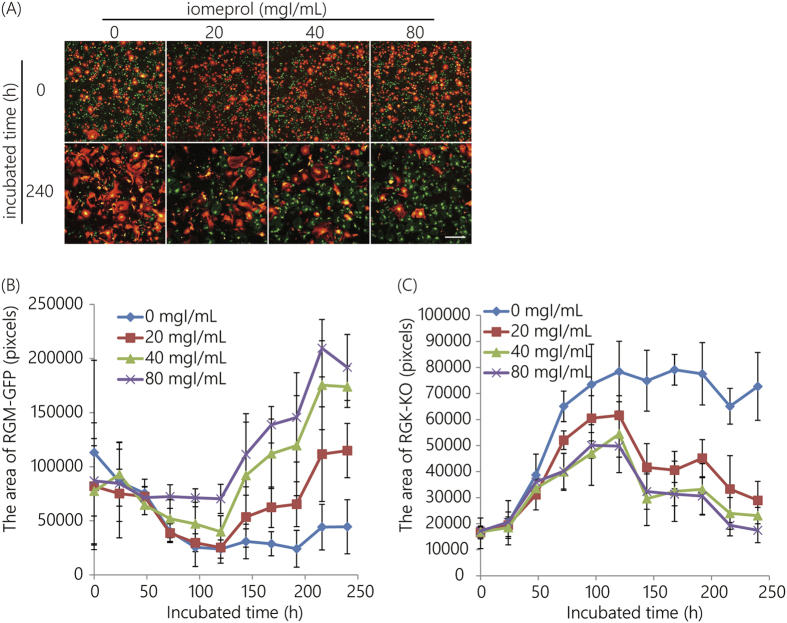
Therapeutic efficacy of irradiation with iomeprol at 10 Gy. (**A**) Fluorescence images after X-ray fluorescence treatment. Scale bar indicates 500 μm. (**B**) The area covered by RGM-GFP cells. (**C**) The area covered by RGK-KO cells. Error bars represent the standard deviation.

**Figure 5:**
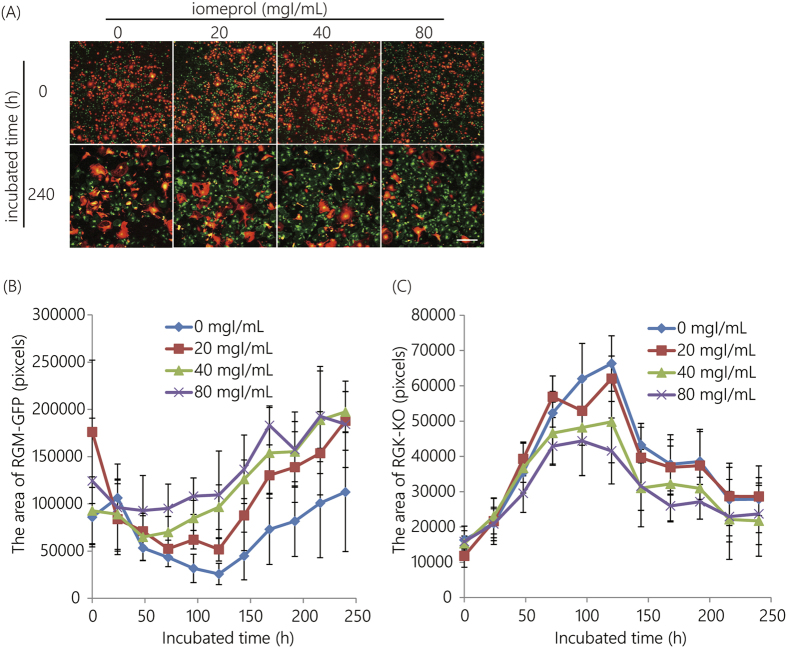
Therapeutic efficacy of irradiation with iomeprol at 20 Gy. (**A**) Fluorescence images after X-ray fluorescence treatment. Scale bar indicates 500 μm. (**B**) The area covered by RGM-GFP cells. (**C**) The area covered by RGK-KO cells. Error bars represent the standard deviation.

Co-cultured cells were exposed to 10 Gy and 20 Gy of radiation (Figs 4 and 5, respectively). With a dose of 20 mgI/mL (Fig. 4B), the area covered by RGM-GFP cells decreased until 125 h, similar to the result observed in Fig. 3B; the decrease in the slope of the curve was weaker with 40 mgI/mL. With 80 mgI/mL the decrease in the slope of the curve was almost abolished. After 125 h of incubation time, slopes of all the curves, with the exception of iomeprol dose 0 mgI/mL, increased. The increments with increasing concentrations of iomeprol were significant. On the other hand, the results for the RGK-KO cell line were contrasting (Fig. 4C). Until 125 h of incubation time, the 0 mgI/mL curve slope demonstrated a consistent increase, similar to that shown in Fig. 3C. The increment rate decreased with 20, 40, and 80 mgI/mL. Following 125 h of incubation time, slopes of all the curves, with the exception of the iomeprol dose 0 mgI/mL, showed a significant decrease.

Even without iomeprol, irradiation at 10 Gy induced minimal RGK-KO cell death after 125 h incubation time (Fig. 4C). However, with 20 Gy, a significant increase in of the number of RGM-GFP cells (Fig. 5B) was observed after 125 h incubation time. On the other hand, a significant decrease in the number of RGK-KO cells (Fig. 5C) was observed.

It must be noted, that X-ray irradiation at 10 Gy with 20 mgI/mL of iomeprol would result in an effect similar to that of irradiation at 20 Gy without iomeprol. The radiation effects were enhanced by increasing the concentration of iomeprol up to 40 mgI/mL (Figs 4 and 5).

### X-ray fluorescence treatment *in vivo*

**Figure 6:**
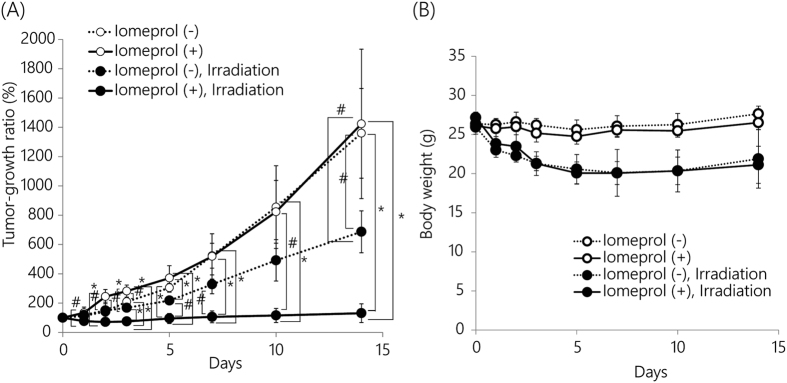
Tumor growth after X-ray fluorescence treatment. (**A**) Tumor growth curve. Iomeprol-injected mice or mice that received irradiation treatment without iomeprol were used as controls. Irradiation at 7.5 Gy was performed with or without iomeprol through aluminum filters (thickness, 2 mm) and copper filters (thickness, 1 mm). Tumor volume (cm^3^) was calculated using the following formula: Tumor volume (cm^3^) = 0.5 × long path (cm) × [short path (cm)]^2^. (**B**) Body weight measurements. Error bars represent the standard deviation (N = 4).

Finally, we demonstrated the utility of this fluorescence treatment *in vivo* (Fig. 6). Effects of the fluorescent X-ray was examined by an injection directly into the tumor avoiding the effect of body distribution of iomeprol as possible. The body weight of iomeprol-treated mice decreased after the radiation treatment; the weight increased after 5 days. The side effects were not severe. All mice survived until 14 days post-treatment. The tumors shrunk in the mice that received both iomeprol and radiation treatment. These results indicated that X-ray fluorescence treatment might enhance the effect of radiation treatment *in vivo*.

## Discussion

The present study indicated that iomeprol could be used as a radiosensitizer. The X-ray fluorescence treatment showed cancer cell-specific cytotoxicity (Figs 4 and 5). We believe that although the X-ray fluorescence attacks the cells directly, it may generate free radicals. Free radical-mediated cell death would target cancer cells rather than normal cells^13^. For instance, oncogenic Ras and hyperactive PI3K/Akt signaling interact in the following manner: free radicals inhibit PTEN; this inhibition enhances Akt activity; and this Akt activity inhibits FOXOs. Thus, the expression of antioxidants such as SOD2, catalase, and sestrin 3 is attenuated. If the levels of free radicals in the cancer cells are elevated, that can result in cell death.

The iodine atoms spherically emit L- and K-band X-rays, with travel distances of approximately 0.1 mm and 17 mm, respectively. The damage from IL-band X-rays occurs locally; however, they are associated with very high levels of toxicity. On the other hand, the travel distance of the Iκ-band is an advantage in X-ray fluorescence treatment in humans because the injury induced by the fluorescence occurs around the tumor tissue. We examined the accumulation of iomeprol, which was mainly located in the tumor following the injection of iomeprol for 3 h (Supplementary Fig. S1). Thus, we would expect to observe iomeprol in the leg tumor that received radiation treatment in the animal study (Fig. 6). Inconveniently, almost the entire mouse body was included within the travel distance of K-band X-ray. Therefore, the body weights of the mice would be decreased owing to radiotoxicity (Fig. 6), although the dose rate here was considered to be milder than that with the single aluminum plate filter (Supplementary Fig. S2). Dose rates were approximately 3.5 Gy/min with a single aluminum filter and 0.7 Gy/min with the aluminum and copper filters. There were no differences in radiotoxicity between these two conditions, and there were no dose rate effects.

Travel distance of the incident X-ray in present study is long compared as that with an ordinary condition such as X-ray irradiation with aluminum filter (2.5 mm thick). The incident X-ray by the aluminum filter is attenuated 0.592 fold by passing through 2 cm of water. On the other hand, incident X-ray with the conditions in present study, which is combined with copper of 1 mm thick and aluminum of 2 mm thick, is attenuated 0.724 fold by passing through 2 cm of water. This incident X-ray is attenuated 0.616 fold even if by passing through 3 cm of water^7^. It is caused by passing through a copper filter which can attenuate X-ray of less than 31 keV, as described in a section of introduction. Although the time for X-ray irradiation by reach necessary exposure dose is prolonged, isodose area can be able to create in a deep tissue under lower side effects.

X-ray fluorescence treatment could be performed using a CT scanner, as reported by a previous study^10^. It would be applicable for transcatheter arterial chemoembolization for hepatoma^14^,^15^,^16^. Chemoembolization starves the hepatoma cells via an ester-binding solution, consisting of poppy oil and an iodine agent, which is added into the hepatic artery through a catheter. It is difficult to achieve complete cure with chemoembolization, and although almost of hepatoma cells appear to undergo cell death, the remaining hepatoma cells can cause recurrence. Complete response is very difficult even if the chemoembolization performed with anticancer drug of cisplatin or doxorubicin^17^,^18^. Theoretically, X-ray fluorescence treatment can be performed with lipiodol. L- and K-band X-rays from iodine should injure around the tissue as described above. If X-ray fluorescence treatment is combined with chemoembolization, it would induce more cell death in the remaining hepatoma cells because the treatment effects occurs by the chemoembolization, drug treatment, and radiotherapeutics effects. As a result, a possibility of complete response would increase.

Other high-Z-elements are also expected to be radiosensitizers. For example, the K-edge of gadolinium is 50.2 keV, and the X-ray fluorescence from gadolinium includes a Kα line (42.7 keV) and an L-band (7–8 keV). Additionally, gadolinium compounds can be injected into humans; for example, texaphyrin could be administrated to patients with incurable cancers in 1998 19. In a future study, we confirm the co-administration of iodine and gadolinium to develop a more effective radiotherapy.

X-ray fluorescence treatment could be applied through the addition of copper and aluminum filters to the X-ray apparatus, and an injection of iomeprol. The cytotoxicity after X-ray treatment without iomeprol at 20 Gy and that after a fluorescent X-ray treatment with 20 mgI/mL of iomeprol at 10 Gy were similar (Figs 4 and 5). Additionally, cytotoxicity after a fluorescent X-ray treatment could be enhanced with increasing the concentration iomeprol up to 40 mgI/mL (Figs 4 and 5). In animal study, an inhibition of tumor-growth ratio after X-ray treatment with iomeprol was approximately twice as much as that without iomeprol compared with non-treatment (Fig. 6). According to these results, fluorescent X-ray therapy would need less than half of exposure dose to show the similar therapeutic effects by ordinary X-ray treatment. We expect this approach to serve as an effective radiation therapy.

## Conclusion

Fluorescent X-ray emission in iomeprol-treated tumor-bearing mice was a more effective treatment than radiation treatment without iomeprol.

## Additional Information

**How to cite this article:** Tamura, M. *et al*. Radiotherapy for cancer using X-ray fluorescence emitted from iodine. *Sci. Rep.* **7**, 43667; doi: 10.1038/srep43667 (2017).

**Publisher's note:** Springer Nature remains neutral with regard to jurisdictional claims in published maps and institutional affiliations.

## Supplementary Material

Supplementary Information
