# Supplementary material for: Radiotherapy for cancer using X-ray fluorescence emitted from iodine
Source: Sci Rep. 2017 Mar 2;7:43667. doi: 10.1038/srep43667 (PMC5333624; doi:10.1038/srep43667)
Supplement: Supplementary Information [file srep43667-s1.docx]

**Supplementary data**

**Radiotherapy for cancer using X-ray fluorescence** **emitted from iodine**

Masato Tamura, Hiromu Ito, Hirofumi Matsui^*^

Faculty of Medicine, University of Tsukuba, 1-1-1 Ten-noudai, Tsukuba, Ibaraki 305-8573, Japan

Corresponding author*:

Hirofumi Matsui, M.D., Ph.D.

Tel: +81-29-853-3218

Fax: +81-29-853-3218

E-mail: hmatsui@md.tsukuba.ac.jp


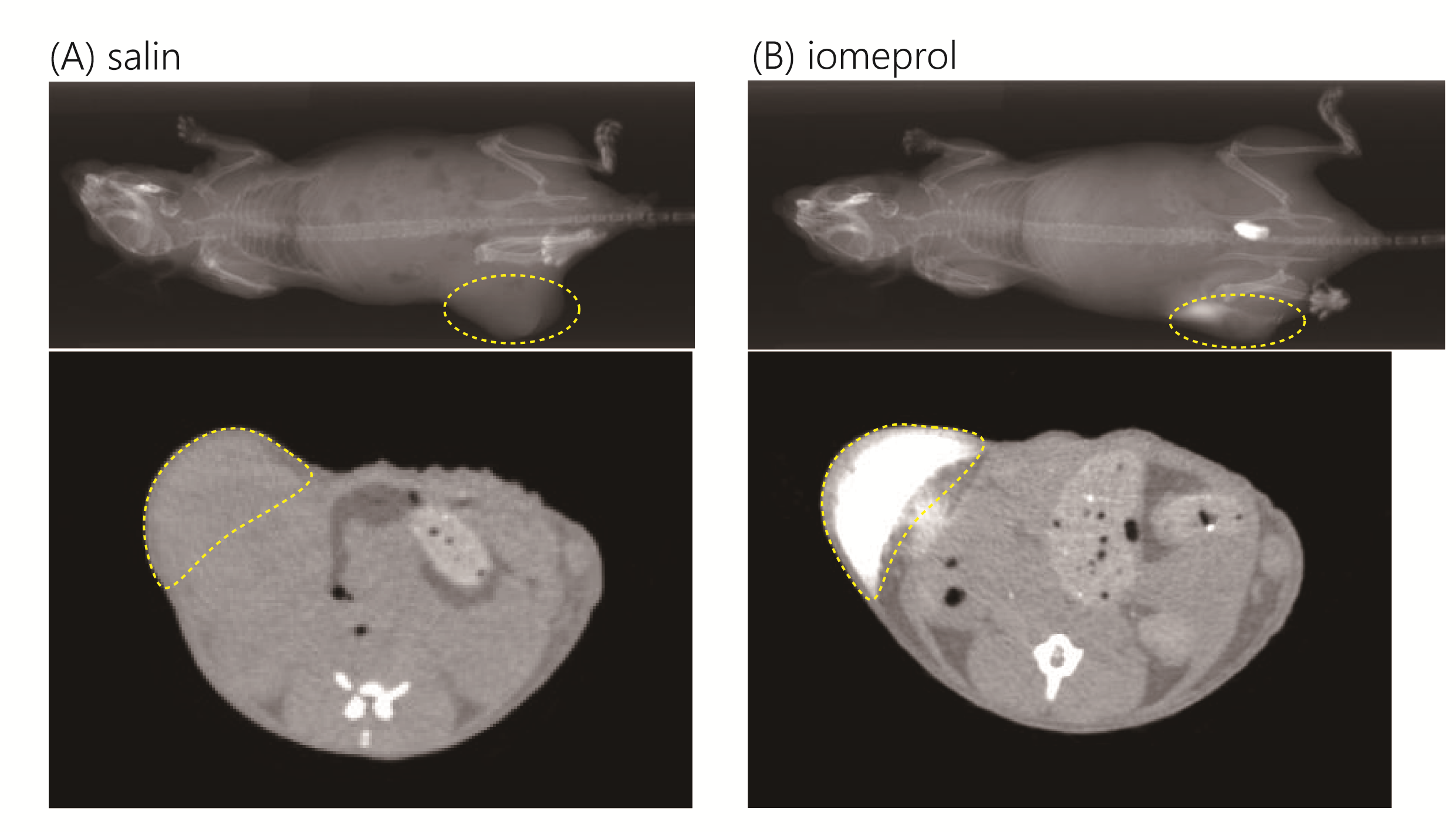


Figure S1. CT scan after an injection of iomeprol with local administration to tumor. The mice were pictured after 3 h from the injection. Yellow dotted line indicates tumor area. CT images were captured using X-ray CT (LaTheta LCT-100, Aloka Co., Tokyo, Japan) (1 slice/ mm).


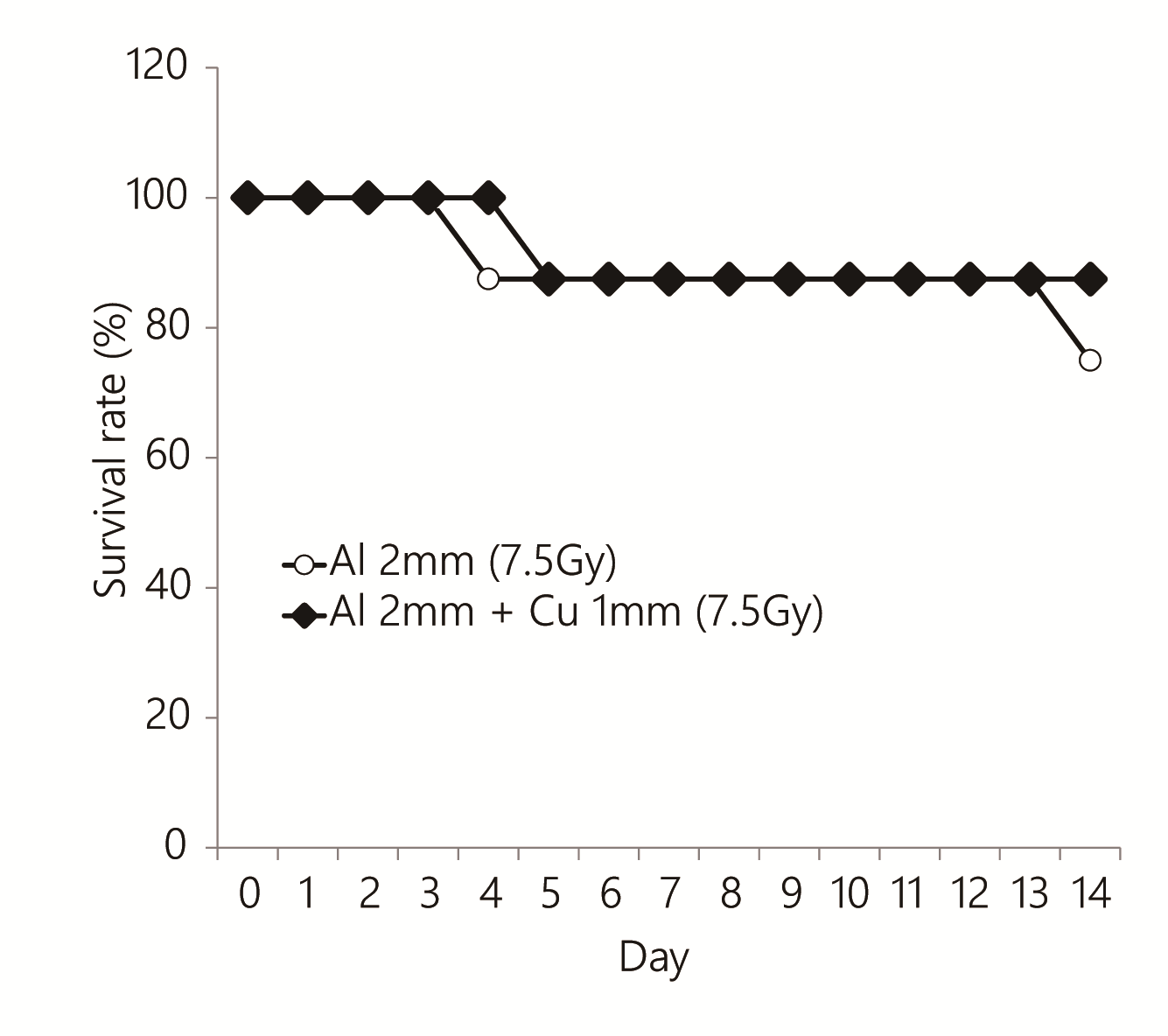


Figure S2. Survival rate after X-ray irradiation. X-ray filters were 2 mm-thickness of aluminum and a two-layer plate of these. (N = 8)
